# Evidence for the Beneficial Effect of Reward on Working Memory: A Meta-Analytic Study

**DOI:** 10.3390/jintelligence12090088

**Published:** 2024-09-11

**Authors:** Weiyu Wang, Xin Yan, Xinyu He, Jiehui Qian

**Affiliations:** Department of Psychology, Sun Yat-sen University, #132 Waihuan Dong Road, Panyu District, Guangzhou 510006, China

**Keywords:** reward, working memory, motivation, meta-analysis

## Abstract

Rewards act as external motivators and can improve performance in various cognitive tasks. However, previous research demonstrated mixed findings regarding the effect of reward on working memory (WM) performance, and the question of whether reward enhances WM performance is arguable. It remains unclear how the effect of reward on WM can be influenced by various factors, such as types of reward and experimental paradigms. In this meta-analytic study, we systematically investigated the effect of reward on WM by analyzing data from 51 eligible studies involving a total of 1767 participants. Our results showed that reward robustly enhanced WM performance, with non-monetary rewards inducing more benefits than monetary rewards. This may be because, while both types of reward could induce extrinsic motivation, non-monetary rewards enhanced intrinsic motivation while monetary rewards reduced it. Notably, all three reward methods—reward binding, reward expectation, and subliminal reward—effectively improved WM performance, with the reward binding paradigm exhibiting the greatest effects. This finding suggests that the reward effect can be attributed to both increasing the total amount of WM resources and improving the flexibility of resource reallocation. Moreover, the type of WM, the experimental paradigms, and the outcome measures are three moderators that should be jointly considered when assessing the reward effects on WM. Overall, this meta-analytic study provides solid evidence that reward improves WM performance and reveals possible mechanisms underlying these improvements.

## 1. Introduction

Working memory (WM) is a short-term, flexible storage system for manipulating information. It plays an essential role in human adaptive behavior and has been a central focus in cognitive psychology research. Extensive studies have shown that there is a fixed limit on the amount of information that can be stored (Bays and Husain 2008; Miller 1956; Schneegans and Bays 2016; van den Berg et al. 2012). However, it is worth noting that the WM capacity is not rigid; it varies among individuals and can be altered by various factors, such as attention (Fang et al. 2023; Kotyusov et al. 2023; Li et al. 2021; Qian et al. 2020), familiarity (Brady et al. 2016), motivation (Krawczyk et al. 2007; Kawasaki and Yamaguchi 2013), etc.

Recent studies have shown that motivation can boost performance across various cognitive domains, including motor control and WM (Codol et al. 2020; Manohar et al. 2019). Reward, as a prevalent source of motivation, has been shown to be effective in this regard. Reward refers to a positive or desirable outcome that follows a behavior, which can be a tangible object, such as food or money, or an intangible experience, such as praise or social approval (Berridge and Kringelbach 2015). Recently, research investigating the influence of reward on WM has gained increasing attention. A wealth of studies has found that reward improves WM performance (Gilbert and Fiez 2004; Rowe et al. 2008; Beck et al. 2010; Marquand et al. 2011; Gaillard et al. 2019; Sandry and Ricker 2020). There are two common ways of manipulating reward. In some studies, participants were required to memorize multiple targets within a single trial, with differential reward assignments. For instance, participants were informed of target values prior to stimuli presentation, e.g., a target on the upper-left quadrant of the screen had a value of five points while others were worth one point (Zheng et al. 2022), resulting in better memory recall for targets with higher rewards. In other studies, a higher reward was assigned to certain trials, e.g., participants were informed at the onset of a trial that correct recognition on this trial could gain ‘100’ or ‘30’ reward points (Sanada et al. 2013). On trials with higher rewards, task performance could be better enhanced compared to low- or non-reward trials.

Researchers have proposed two potential explanations to account for how reward enhances WM performance: increasing the attentional priority for processing or augmenting the total amount of resources for processing. On the one hand, for studies with varying reward levels within the same trial, rewards may function as motivational incentives, guiding the deployment of cognitive resources to effectively orient attention and prioritize the processing of task-relevant information (Engelmann et al. 2009; Pessoa 2009; Pessoa and Engelmann 2010; Watanabe 2007). Allen and Ueno (2018) demonstrated that during the encoding phase, stimuli with higher rewards were likely to lead to higher resource concentration, and the degree of attention deployment could be controlled by the value of the reward. On the other hand, in studies manipulating rewards across different trials, rewards may enhance the total amount of resources available for WM in the rewarded trials. The resource rational theory of WM (van den Berg and Ma 2018) challenges the conventional view of a fixed WM capacity. Their theory posits that neural coding is costly (Lennie 2003; Sterling 2017), prompting the brain to trade the behavioral benefits of high memory precision against the cost of the memory resources invested in stimulus encoding. Consequently, it is the cost of reducing WM errors that ultimately constrains WM performance, suggesting that rewards can offset this cost and thus improve WM performance. In previous studies that employed rewards, participants were often strongly motivated to invest more cognitive effort and fully deploy resources to offset the cost of reducing memory errors, thereby improving the performance of WM. Indeed, several studies have shown that individuals can improve their WM performance when incentivized (Gilbert and Fiez 2004; Kawasaki and Yamaguchi 2013; Sanada et al. 2013). However, these studies either did not find or did not report any associated changes in reaction times, making it hard to tell whether the observed improvements represent genuine improvements or simply reflect a trade-off with processing speed.

Although the notion that rewards can improve WM performance is commonly held, there remains a body of research showing contradictory findings. Some studies failed to observe a significant effect of reward on WM accuracy (Krawczyk et al. 2007; Hager et al. 2015; Di Rosa et al. 2019). Meanwhile, others found that the influence of reward expectation on WM diminished when the tasks were relatively simple and the WM load was relatively low (Fairclough et al. 2018; Gaillard et al. 2019). The beneficial effects of reward were not consistently reported, with some studies indicating an effect for participants with better WM capacity, while others did not (Thurm et al. 2018; Manga et al. 2020).

To our knowledge, no previous study has systematically and quantitatively synthesized the empirical studies that have investigated whether and how reward can improve performance in WM. Therefore, in the present meta-analysis, we systematically and quantitatively summarized the results of previous studies and examined the nature and extent of reward on WM performance given different influencing factors. In particular, we aimed to address two questions: (a) Whether a reward is effective in improving WM overall and (b) What kind of influencing factors can modulate the effect of reward on WM.

The investigation of the impact of rewards on WM performance holds important theoretical and practical significance. On the one hand, rewards may increase the motivational salience of the task, which might make the task appear more challenging or engaging, thereby potentially influencing task performance. On the other hand, rewards may bias the allocation of cognitive resources and attention control, temporarily affecting one’s WM ability towards certain stimuli (e.g., enhanced ability for the rewarded stimuli and maybe reduced ability for the unrewarded ones), thus influencing one’s WM performance. From a theoretical perspective, it offers insight into the mechanisms underlying the interaction between motivation and cognitive functions, contributing to resolving the ongoing debate regarding the nature of the relationship between the two. Understanding how rewards influence WM performance may inform evidence-based decision-making across various domains, which may have practical implications on work efficiency enhancement and self-fulfillment.

## 2. Possible Moderators of Reward Effects on WM

### 2.1. The Type of Reward

External rewards can be categorized into two types: monetary rewards and non-monetary rewards. Monetary rewards, such as pay increases and bonuses, usually have substantial cash values (Martin and Harder 1994). For example, Sanada et al. (2013) instructed participants to detect changes between two sequentially presented stimuli and they were told that reward points could be earned if their answers were correct, and the final monetary reward was calculated based on the total points acquired. Non-monetary rewards usually take the form of positive feedback in experimental instructions provided by experimenters, which often serve to satisfy socioemotional needs (Martin and Harder 1994). It has been found in a belief updating task that, compared to non-monetary rewards, monetary rewards resulted in a faster learning rate (i.e., the speed of belief updating) and greater changes in neural indicators (e.g., feedback-related negativity and late positive potential; Bennett et al. 2019). In addition, compared to non-monetary rewards, monetary rewards have been found to activate the thalamus more strongly but to activate the amygdala weakly (Rademacher et al. 2010). Given these findings, the two types of rewards may be processed in divergent ways, and it is possible that they have different impacts on WM. For instance, Zheng et al. (2022) used a memory recall task and observed better performance in the monetary reward condition than in the non-monetary reward condition. Therefore, in this meta-analysis, the effects of the two types of rewards on WM were systematically summarized and compared.

### 2.2. Reward Methods

There are three commonly used reward methods to explore the impact of rewards on WM performance. The reward binding method is an approach that involves associating particular stimuli features with rewards. For instance, when memorizing a variety of items, only specific attributes of these items (e.g., certain colors among various colors) are linked with rewards (Morey et al. 2011). As a result, participants tended to allocate more WM resources to these targets that presumably had a high priority, and the recall accuracy for these specific stimuli can be improved (Hitch et al. 2018; Klink et al. 2017).

The reward expectation method is a technique that involves informing participants about the existence or magnitude of the reward at the beginning of a trial (Manga et al. 2020), and the rewards are indiscriminately assigned to every stimulus within the trial. The memory resources are not priorly oriented to particular targets; instead, rewards may act as catalysts, bolstering mental efforts within the trial (van den Berg and Ma 2018; Capa and Bouquet 2018). For example, mnemonic strategies and other higher-order cognitive processes may be selectively adopted based on task demands.

The subliminal reward method is similar to the reward expectation method, except for a key difference: cues about the existence or magnitude of the reward are presented subliminally at the start of the trial. In other words, while both methods involve pairing rewards with all stimuli on one trial, unlike the reward expectation method, the distinguishing feature of the subliminal reward method is that participants are unaware of the reward cues (Xu et al. 2022). As a result, any noticeable improvement in working memory resulting from this method is likely driven solely by unconscious cognitive processes.

### 2.3. The Type of WM

It has long been suggested that visual and verbal stores are two separate, limited-capacity subsystems of the WM model (Baddeley and Hitch 1974; Luck and Vogel 1997; Smith et al. 1996). Verbal WM utilizes subvocal rehearsal to maintain verbal material and is typically assessed using the N-back paradigm (Belayachi et al. 2015; Hager et al. 2015), while visual WM maintains visual material through visualization and is usually evaluated using the change detection paradigm or recall/estimation tasks (Allen and Ueno 2018; Morey et al. 2011). Visual and verbal WM differ in several ways. For example, the capacity of verbal WM (Miller 1956) is commonly considered to be larger than that of visual WM (Luck and Vogel 1997; Cowan 2001). Using a dual-task paradigm, verbal and visual-spatial WM tasks could be performed largely in parallel and the two could be performed simultaneously with few interferences (Baddeley and Hitch 1974). Moreover, there is no significant correlation between performance on visual and verbal WM tasks at the individual level (Shaw et al. 2006). Neurologically, there are distinct cerebro-cerebellar activation patterns in verbal and visual WM. It has been shown that verbal WM activates a cerebrocerebellar circuitry that comprises left-lateralized language-related brain regions, including the inferior frontal and posterior parietal areas and right-lateralized superior and inferior cerebella areas. In contrast, visual WM activates a distributed network of bilateral inferior frontal and inferior temporal areas, as well as bilateral superior and inferior cerebellar areas (Ng et al. 2016). Pessoa (2009) suggested that reward mechanisms can modulate cognitive processes differently depending on the neural substrates involved. Given that visual and verbal WM rely on distinct neural substrates, it is plausible that rewards might influence these systems differently. In summary, considering the differences in capacity, function, and neural substrates between visual and verbal WM, we speculate that rewards may have differential effects on these two types of WM.

### 2.4. Paradigm of WM

Different experimental paradigms serve to investigate various aspects of WM. Two frequently used paradigms for assessing memory performance are recall and recognition tasks. In both tasks, memory items are presented first and participants’ memories are tested after a delay. The main difference lies in the way their memory is being tested. In a recognition task, participants are typically presented with either the item previously shown (target) or another new item not shown before (foil) during the test stage. Participants are then required to identify the target by judging whether the test item is old (previously presented) or new (e.g., Qian et al. 2019, 2020). In a recall task, participants are typically asked to reproduce the target without the target or foils being presented during the test stage (e.g., Zhang et al. 2021).

There can be several variants in the experimental paradigm used in both tasks. For example, retrieval cues can be applied during memory maintenance in both recall and recognition tasks (Fang et al. 2023; Li et al. 2021). For recall tasks, a free recall task indicates that participants are required to recall as many items in memory lists as possible in the absence of any external cues, while in a cued recall task, they are provided with retro-cues to aid memory retrieval. However, in both types of recall tasks, one needs to rely on internal retrieval cues to generate and reproduce the specific value in the tested dimension of the target item. As a result, recall tasks may demand strategic operations, as participants must develop internal strategies to guide the memory search (Stebbins et al. 1999). In comparison, recognition tasks require minimal strategic operations, as participants only need to identify the target among foils (Unsworth and Engle 2007).

The change detection task, which can be considered a variant of the recognition task in the domain of visual WM, was developed by Luck and Vogel in 1997. In this task, participants are presented with a memory display, followed by a test stimulus that may be identical or different. The participant’s task is to judge whether the test display matches the memory display. The estimate of memory capacity K can be calculated by hits and false alarm rates in the task (Pashler 1988; Unsworth et al. 2014), and an individual’s memory capacity K is often correlated with one’s change detection accuracy given a fixed set size of stimuli (see Section 2.5).

Another commonly used task in WM studies is the n-back task (Chen et al. 2008). In the n-back task, stimuli are sequentially presented and participants are asked to report whether the item in the current display matches the item presented ‘n’-display back. Typically, ‘n’ varies across trials to assess the effect of different memory demands upon behavioral performance and physiological correlates (Redick and Lindsey 2013).

In addition, the operation-word-span task (OSPAN) is also an experimental paradigm commonly used to measure WM capacity, in which participants need to carry out a series of simple mathematical operations while they attempt to remember a list of unrelated words (Turner and Engle 1989). By varying the length of the word list, WM capacity for verbal words can be determined.

The delayed estimation task is frequently used for examining the fidelity of visual representations in WM (Bays et al. 2009; Wilken and Ma 2004). In this task, a set of memory items is presented (e.g., color or orientation), and participants must reproduce the target feature value after a brief delay (Ricker et al. 2023). The estimation error is often used as the outcome measure, which is a continuous variable. Various theoretical frameworks have been proposed to model the estimation error, such as the standard mixture model (Zhang and Luck 2008), the joint-representation model (Bae et al. 2015), etc. (Bays et al. 2009; Zhang and Qian 2022), in an attempt to clarify the underlying mechanisms of WM representation.

Considering the distinctions and interconnections among these paradigms, we hypothesize that they may exert a moderating effect on the impact of rewards on WM performance.

### 2.5. Outcome Measures

Typical measures of WM performance include recall error, accuracy, capacity, and reaction time. Depending on different experimental paradigms, different types of measures are preferably used. Error is usually used in memory recall studies that require participants to reproduce the feature value of the target (e.g., orientation of a bar, hue of a color, etc.). It is conventionally calculated as the absolute value of the difference between the actual feature value of the target and the reproduced one. Accuracy is usually used in change detection paradigms that require participants to detect whether there has been a change between the memory display and the test display and is defined as the proportion of correct responses. Capacity is often reported in addition to accuracy, and its calculation is usually based on hit rates and false alarm rates, according to Pashler (1988) and Cowan (2001). Reaction time refers to the amount of time it takes for a person to respond to a specific stimulus, typically measured from the onset of the stimulus to the onset of the response. Generally, smaller errors, higher accuracy rates, higher memory capacity, and a shorter reaction time indicate better WM performance.

Bays and Husain (2008) suggested that memory capacity and accuracy are indications of the maximum amount of information or number of items stored in WM, whereas recall error indicates the fidelity and precision of memory representation; therefore, the two measures reflect two distinct aspects of WM ability. However, the above four measures are not completely independent. Since both accuracy and capacity can be calculated by hit rates and false alarm rates, these two share some common properties and often yield similar result patterns (Li et al. 2018; Qian et al. 2019).

In addition, rewards may have different effects on accuracy/error and reaction time. For example, providing a reward can lead to a reduction in errors, but it may also induce participants to adopt a more cautious approach, leading to longer response times (Grogan et al. 2022). These effects are often known as the cost–benefit trade-off (Shadmehr et al. 2010) and the speed–accuracy trade-off (Fitts 1954). Previous studies have shown that the two kinds of trade-offs change as the value of the reward varies, suggesting a common underlying mechanism in the brain that maximizes the expected utility resulting from the potential reward and the cost (Peternel et al. 2017).

### 2.6. Participant Group

Rewards can enhance WM performance by increasing attention and memory priority or offsetting energetic costs. However, it is important to note that the impact of rewards can vary across different populations. Studies have shown that in the case of elderly individuals (Rhodes and Katz 2017) and individuals with schizophrenia (Barch 2005), the positive effect of rewards diminishes. This divergence in outcomes is likely due to the fact that, in comparison to young adults, older individuals typically exhibit less flexible WM ability and lower memory capacity, making it more challenging for them to benefit from cognitive training. On the other hand, individuals with schizophrenia often show reduced motivation, which can be attributed to abnormalities in their processing of reward. Compared to healthy adults, brain regions relevant to reward processing in schizophrenia tend to exhibit weaker activation in response to monetary reward (Juckel et al. 2006). Based on these findings, we speculate that the participant group may moderate the effect of reward on WM. Compared to healthy young adults, rewards are likely to have a less pronounced effect on the WM performance of both elderly individuals and those with schizophrenia.

## 3. Methods

### 3.1. Literature Search

To locate relevant articles, we first conducted online searches of the Web of Science database, using the keywords *WM* & *reward/rewards*, *WM* & *reward expectation*, and *WM reward* & *motivation*, as well as the Chinese National Knowledge Infrastructure (CNKI), using the same keywords in Chinese. Secondly, we read the titles and abstracts to determine the relevance of each article. These preliminary searches yielded a database of 89 articles covering the years 1981 to 2022.

### 3.2. Criteria for Selection of Studies

The studies were included if they met the following criteria: (1) the participants were human; (2) the study object was WM; (3) the study involved manipulation of rewards or reward expectations; (4) the study provided behavioral data of WM performance, such as accuracy and reaction time; (5) the study used quantifiable variables and was not purely descriptive or anecdotal in nature (such as a case study); and (6) the study should provide the mean, standard deviation (s.d.), sample size, test statistics, or other data that can be calculated for effect size.

Among the 89 articles identified through the literature search, 39 met all of the specified criteria and were included in the meta-analysis. The remaining 50 articles were excluded for various reasons, including 14 that involved animal participants, 10 that did not manipulate rewards or reward expectations, 21 where the study did not investigate WM (e.g., they investigated long-term memory or source memory), and 5 due to lack of sufficient information to estimate effect sizes, such as missing standard deviations or incomplete statistical outcomes.

### 3.3. Coding Procedures

Three of the authors coded the following information from the included studies: (1) basic information about the research, including author(s), journal (or dissertation), and publication year; (2) demographic information, including the sample size, mean age, sex, and types of participants; (3) study characteristics, including types of reward, reward level, paradigms, types of WM, outcome measures, and experimental design; and (4) statistical results used for estimating effect sizes, including the mean and s.d. for the experimental conditions, the *t*-test, F test, or other statistics, and *p*-values. To ensure coding reliability, the three coders cross-checked all the coding sheets for each study.

### 3.4. Moderator Variables

Due to the divergence in the experimental design and the stimuli used in the various studies, moderator analysis was necessary to examine the possible factors influencing the effect of reward on WM performance. Six moderators were taken into consideration: (1) type of reward: rewards were coded as two main types: monetary rewards and non-monetary rewards; (2) reward methods: the reward methods were classified into three categories: reward binding, reward expectation, and subliminal reward; (3) type of WM: visual WM and verbal WM; (4) paradigm of WM: five paradigms were coded: n-back, change detection, recognition, recall, and delay estimation. It is important to note that there was one study using the OSPAN paradigm and two studies whose paradigms did not fit into either of these categories, so we categorized them as ‘others’; (5) outcome measures: three outcome measures were coded: error, reaction time (RT), and accuracy (ACC). It is worth noting that one study did not report accuracy and only reported capacity. Since the calculation of capacity was based on accuracy, we combined the effect size of capacity in this study into the category of accuracy. To ensure the independence of effect sizes, when a study contributed more than one effect size using different outcome measures, we assigned the effect size of that study to the group with a smaller total number of studies; and (6) participant group: although participants included in most of the included studies were healthy young adults, there were a small number of elderly, schizophrenic, and ADHD participants. Due to the relatively small number of studies involving these participants and to avoid a potential low statistical power when conducting moderator analysis, participants were categorized into two groups: healthy young adults and others.

### 3.5. Data Analysis

The standardized mean difference, Cohen’s d, was used as the measure of effect size. Cohen (1988) proposed that a value of d of 0.2, 0.5, or 0.8 can be considered as a small, moderate, or large effect size, respectively. Since we hypothesized that rewards would enhance WM performance, effect sizes were given positive signs when they were consistent with this expectation, and negative signs when they were not. When means and s.d. were available, effect sizes were calculated using the formula given by Cohen. When an inferential statistic (typically the *t*-test, *p*, r, or F) was available, the formula provided by Lipsey and Wilson (2001) was used. When a study provided only a significance level and did not provide data that could be used to calculate the effect size, the minimum d value that would enable the study to reach that significance level was calculated as the effect size for the study. When a study’s *p*-value was not significant but no specific *p*-value was provided, the effect size was coded as 0 (Kalma 1991).

To ensure the independence of effect sizes, each independent sample was coded only once to produce one effect size. When a study produced multiple effect sizes, their combined effect sizes were calculated using weighted averages.

The Stouffer method of adding standard normal deviation zs was used to estimate significance levels for combined studies (Mosteller and Bush 1954). In addition to computing combined effect sizes and probability levels, a fail-safe N was also computed for each analysis (Rosenthal 1979). The fail-safe N is an estimate of the number of unpublished, non-significant studies that would have to exist for the obtained probability value to be rendered non-significant. Publication bias was also assessed using trim-and-fill analysis.

Comprehensive Meta Analysis Version 3.3.070 (CMA, 3.0) was used to conduct the data analysis. A mixed-effects model implemented in CMA 3.0 was used to analyze moderating effects.

## 4. Results

### 4.1. The Overall Effect

This meta-analysis was conducted on 39 articles. Effect sizes were obtained from 55 studies with independent samples, with a total of 1802 participants. Each sample size ranged from eight to 146. The characteristics of these studies are summarized in Table 1.

**Figure 1:**
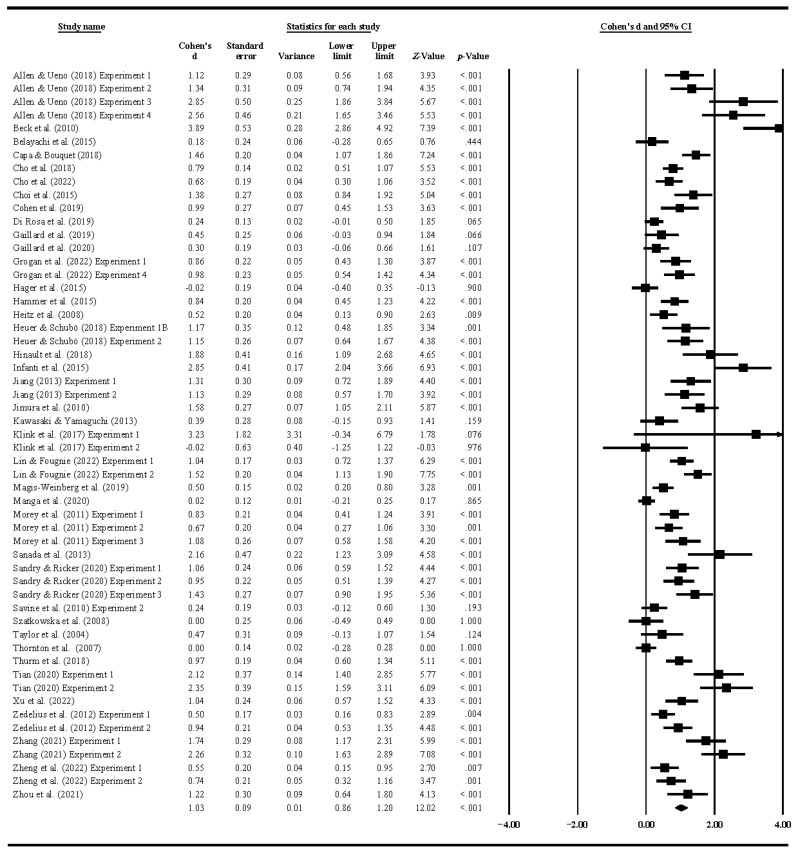
Effect sizes for all studies.

The results of this analysis revealed a mean estimated Cohen’s d of 1.03 with 95% CI = [0.86–1.20], which could be considered a fairly large effect. The Stouffer method of significance testing showed that reward significantly improved WM performance, *p* < 0.001 (Figure 1).

### 4.2. Publication Bias

**Figure 2:**
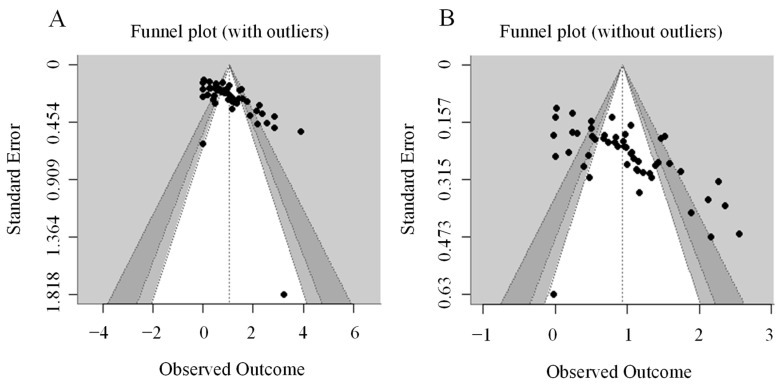
Funnel plot. (**A**) Funnel plot (with outliers). (**B**) Funnel plot (without outliers).

We used a trim-and-fill analysis to assess the publication bias. As the funnel plot (Figure 2A) has shown, the majority of effect sizes were well-balanced around the mean effect size, except for four studies that had large effects (d > 2.8). We suspected that the exceedingly large effect size might result from individual differences and large sampling errors. Therefore, we performed the Kolmogorov–Smirnov normality test and stem-leaf plot analysis, which showed that the normality hypothesis did not hold (*p* = 0.008) and that there were four extremes of Cohen’s d. After removing the extreme values, the Shapiro–Wilk test (N > 50) was performed again, and the results showed adherence to the assumption of normality (*p* = 0.200). As shown in the updated funnel plot (Figure 2B), the remaining studies were evenly distributed, with appropriate sample sizes and relatively good quality. The remaining 51 studies were then used as the total sample for moderator analyses, with a total of 1711 participants.

Results revealed a mean estimated Cohen’s d of 0.93 with 95% CI = [0.77–1.08], which was a fairly large effect. Null hypothesis significance testing showed that reward significantly improved WM performance, *p* < 0.001 (Table 2). The test of heterogeneity was significant, Q(50) = 314.88, *p* < 0.001, suggesting that the effect sizes varied significantly across different studies. Examining the potential moderators might therefore clarify the factors that account for this variability. A file drawer analysis or fail-safe N indicated that 9362 missing or non-published studies that averaged null findings would have to exist to cancel this effect. However, the existence of so many studies was unlikely given the number of the included studies in the current meta-analysis and therefore the current result can be considered to be robust.

### 4.3. Moderator Analyses

**Figure 3:**
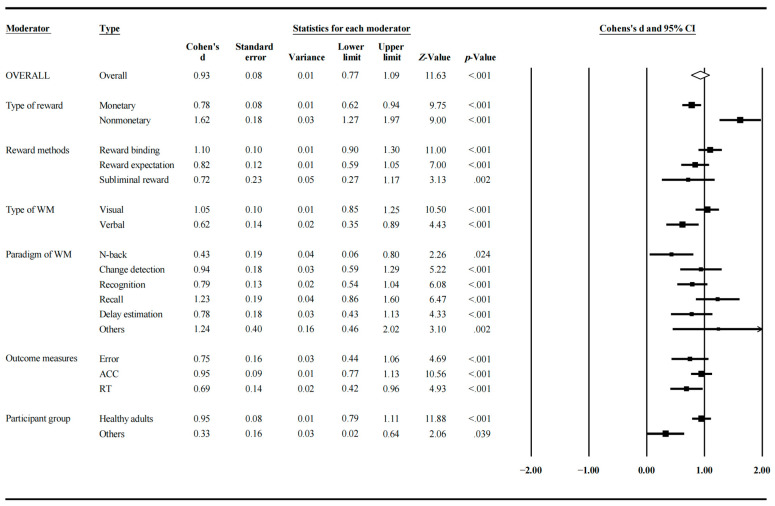
Forest plots for all moderators.

The results of moderator analyses are presented in Table 2 and Figure 3. Overall, we found that the type of reward, reward methods, the type of WM, paradigms of WM, outcome measures, and participant group influenced the effect of reward on WM.

#### 4.3.1. The Type of Reward

Thirty-nine studies used monetary rewards and the other ten studies used non-monetary ones. The homogeneity test showed that the between-subgroup variance was significant (Q(1) = 19.10, *p* < 0.001). Specifically, if the reward was monetary, the effect size was moderate (d = 0.78, *p* < 0.001); if not, the effect size was large (d = 1.63, *p* < 0.001). File-drawer analysis showed that fail-safe N was 4440 for the monetary reward group and 713 for the non-monetary reward group.

#### 4.3.2. Reward Methods

Twenty-three studies employed the reward binding method, twenty-eight utilized the reward expectation method, and only four applied the subliminal reward method. The homogeneity test was marginally significant (Q(2) = 4.27, *p* = 0.10), suggesting a trend that variance differed significantly across different subgroups.

Specifically, when the reward binding method was used, the effect size was large (d = 1.10, *p* < 0.001). In contrast, for both reward expectation (d = 0.82, *p* < 0.001) and subliminal reward (d = 0.72, *p* = 0.002) methods, the effect size was moderate. In other words, WM performance showed the greatest improvement when using the reward binding paradigm, followed by the reward expectation paradigm, and the least pronounced effect was found for the subliminal reward paradigm. File-drawer analysis showed that fail-safe N was 2581 for the reward binding group, 2307 for the reward expectation group, and 59 for the subliminal reward group.

#### 4.3.3. Type of WM

Thirty-seven studies focused on measuring visual WM, while fourteen studies assessed verbal WM. The homogeneity test showed that the between-subgroup variance was significant (Q(1) = 6.80, *p* = 0.009). For visual WM, the effect size was large (d = 1.05, *p* < 0.001); whereas for verbal WM, the effect size was moderate (d = 0.62, *p* < 0.001). File-drawer analysis showed that fail-safe N was 5712 for the visual WM group and 433 for the verbal WM group.

#### 4.3.4. Paradigm of WM

Seven studies employed n-back tasks, six used change detection tasks, thirteen used recognition tasks, ten utilized recall tasks, eight used delay estimation tasks, and three other studies were categorized as other paradigms. The homogeneity test showed that the between-subgroup variance was marginally significant (Q(5) = 10.42, *p* = 0.064). For studies that used n-back tasks, the effect size was small (d = 0.43, *p* = 0.025); whereas for studies that employed change detection (d = 0.94, *p* < 0.001), recognition (d = 0.79, *p* < 0.001), recall (d = 1.23, *p* < 0.001), and delay estimation (d = 0.78, *p* < 0.001), the effect size was large. For studies that used other paradigms, the effect size was larger (d = 1.24, *p* = 0.002). File-drawer analysis showed that fail-safe N was 46 for the n-back task group, 118 for the change detection task group, 477 for the recognition task group, 508 for the recall task group, 240 for the delay estimation task group, and 52 for the other task group.

#### 4.3.5. Outcome Measures

Eight studies utilized estimation error as the outcome, 42 studies employed accuracy as the outcome, and 20 studies adopted reaction time as the outcome. The homogeneity test was not significant (Q(2) = 3.10, *p* = 0.212), suggesting that variance differed significantly across different subgroups.

Specifically, if accuracy was used as the outcome, the effect size was large (d = 0.95, *p* < 0.001); if error (d = 0.75, *p* < 0.001) or reaction time (d = 0.69, *p* < 0.001) was used, the effect size was moderate. The result showed that all three outcomes could effectively capture the beneficial effect of reward on WM. File-drawer analysis showed that fail-safe N was 250 for the error group, 6265 for the accuracy group, and 806 for the response time group.

#### 4.3.6. Participant Group

Forty-nine studies involved healthy young adults as participants while six studies did not. The homogeneity test showed that the between-subgroup variance was significant (Q(1) = 11.40, *p* = 0.001). For studies with healthy young adults, the effect size was large (d = 0.95, *p* < 0.001); otherwise, the effect size was small but significant (d = 0.33, *p* = 0.047). File-drawer analysis showed that fail-safe N was 8503 for the healthy adults and 23 for the others.

#### 4.3.7. Secondary Subgroup Analysis

Because the heterogeneity (Q) remained high in several subgroups even after moderator analyses, we performed secondary subgroup analyses to further reduce within-group variances and to account for variances that may influence the reward effects. To control for Type-I errors that potentially resulted from small sample sizes (insufficient number of studies), we chose four subgroups that had large Qs and sufficient numbers of studies to perform further subgroup division (Table 3).

First, we performed a secondary subgroup analysis for the monetary reward subgroup (Q = 285.13) based on outcome measures. Out of 42 studies that employed monetary rewards, eight studies utilized error as the outcome, 19 studies used accuracy, and 15 studies used reaction time. The homogeneity test was marginally significant (Q(2) = 5.66, *p* = 0.06), suggesting a trend that variance differed significantly across different subgroups. Specifically, if the outcome measure was accuracy (d = 0.89, *p* < 0.001) or error (d = 0.86, *p* < 0.001), the effect size was large; if reaction time (d = 0.48, *p* < 0.001) was used, the effect size was moderate. File-drawer analysis showed that fail-safe N was 247 for the error group, 1230 for the accuracy group, and 227 for the response time group.

Second, a secondary subgroup analysis was performed for the visual WM subgroup (Q = 296.60) based on outcome measures. Out of 38 studies that focused on visual WM, seven studies utilized error as the outcome, 21 studies employed accuracy, and 10 studies used reaction time. The homogeneity test was marginally significant (Q(2) = 6.47, *p* = 0.039). If the outcome measure was reaction time (d = 0.75, *p* < 0.001) or error (d = 0.79, *p* < 0.001), the effect size was large; if accuracy (d = 1.24, *p* < 0.001) was used, the effect size was much larger. File-drawer analysis showed that fail-safe N was 252 for the error group, 2006 for the correct/capacity group, and 170 for the response time group.

Third, we performed two separate secondary subgroup analyses for the reward binding (Q = 55.43) and reward expectation (Q = 145.74) subgroups based on the type of reward. Out of 23 studies that employed the reward binding method, 15 used monetary rewards and the other eight studies used non-monetary ones. The homogeneity test showed that the between-subgroup variance was significant (Q(1) = 5.59, *p* = 0.018). Specifically, the effect size was large (d = 1.03, *p* < 0.001) if the reward was monetary, and it was even larger (d = 1.53, *p* < 0.001) if the reward was non-monetary. File-drawer analysis showed that fail-safe N was 1155 for the monetary reward group and 400 for the non-monetary reward group.

On the other hand, out of 22 studies that employed the reward expectation method, 20 used monetary rewards and only two studies used non-monetary ones. The homogeneity test showed that the between-subgroup variance was significant (Q(1) = 38.06, *p* < 0.001). Interestingly, the effect size was small (d = 0.52, *p* < 0.001) if the reward was monetary, and it was fairly large (d = 1.99, *p* < 0.001) if the reward was non-monetary. File-drawer analysis showed that fail-safe N was 512 for the monetary reward group.

## 5. Discussion

Pulling from the results of 51 studies with a total of 1711 participants, we found robust evidence that reward enhances WM performance. This effect varied based on the type of reward or reward method employed, and it was also moderated by factors such as the type of WM, paradigms of WM, various outcome measures, and participant groups.

**The type of reward**. Interestingly, we found that the effect of monetary reward on WM is weaker than non-monetary reward. Fail-safe N reveals robustness in these results. This finding contradicts the conclusion of some studies, which suggest that monetary rewards lead to faster learning and thus a greater facilitation effect (e.g., Bennett et al. 2019). We propose several possible reasons for these results. Firstly, monetary rewards may induce excessive motivation to achieve optimal performance. As Botvinick and Braver (2015) have suggested, strong incentives can sometimes induce decrements in performance, a phenomenon referred to as “choking under pressure”. Several studies have demonstrated that high monetary rewards can result in less than optimal performance across various tasks, including motor learning, intelligence tests, and long-term memory (Kuhbandner et al. 2016; Cheng et al. 2020). It has also been found that individuals pursuing monetary rewards often experience heightened feelings of fear (Castro et al. 2021), whereas non-monetary social rewards have been associated with increased positive emotions (Lee et al. 2011). Neuroimaging studies have shown stronger thalamus activation to monetary rewards than non-monetary social rewards, where the thalamus is suggested to modulate goal-directed behavior through ranking incentive values (Rademacher et al. 2010). In other words, activation in the thalamus may reflect subjective reward value, and monetary rewards can be considered to have a greater value than social rewards. Therefore, we suspect that for WM specifically, the motivation brought by a non-monetary reward is optimal for performance while that brought by a monetary reward might be excessive.

Secondly, it is possible that both types of reward could induce extrinsic motivation, while the two may have different impacts on participants’ intrinsic motivation. Extrinsic motivation is often considered as a catalyst for action that is driven by external rewards, such as money, grades, etc., and intrinsic motivation is the incentive to complete a task to satisfy one’s psychological needs, such as competence, enjoyment, etc. Deci (1971) observed that using money as an external reward decreased intrinsic motivation (i.e., monetary reward reduced intrinsic motivation), whereas verbal reinforcement and positive feedback tended to enhance it (i.e., non-monetary reward enhanced intrinsic motivation). The introduction of money into an activity often prompts individuals to reassess their motivations, shifting from intrinsic to extrinsic, thereby diminishing their inherent drive (Lepper and Greene 1976). Conversely, social approval, which mostly triggers intrinsic motivation, does not typically have the same effect. Previous studies have shown that intrinsic motivation is better at promoting individual performance in self-regulated learning and employee outcomes (Young 2005; Moos 2010; Kuvaas et al. 2017). It is possible that in the field of WM, intrinsic motivation also plays a more pivotal role compared to extrinsic motivation. Consequently, the use of monetary rewards resulted in less improvement when compared to non-monetary rewards.

One might speculate whether the difference in the effects between monetary and non-monetary rewards was due to unequal sample sizes, since there were 39 studies using monetary rewards and only 10 studies using non-monetary rewards. To test this possibility, we performed a Bootstrap analysis for both monetary and non-monetary studies and randomly sampled the effect sizes from 10 studies from each type of study with 10,000 resamples. The Bootstrapped mean effect size was 0.98 for the monetary studies and 1.80 for the non-monetary ones. In other words, the effect size of the non-monetary studies was significantly larger than that of the monetary studies, ruling out the confounding of unequal sample sizes. However, one confounding factor remains unresolved. Most of the studies utilizing non-monetary rewards employed the reward binding method, which was shown to have the largest effect among the three reward methods examined in this meta-analysis. To further clarify the possible confounds, secondary subgroup analyses were conducted and the results are discussed in the following section.

**Reward methods.** We found a trend that the reward binding method induced a larger effect compared to both reward expectation and subliminal reward methods. This result was consistent with empirical findings showing that studies applying the reward binding method consistently showed a reward enhancement effect on WM (Gong and Li 2014; Klink et al. 2017; Klyszejko et al. 2014; Christie and Schrater 2015; Wallis et al. 2015), while studies using the reward expectation method have yielded mixed results (Gilbert and Fiez 2004; Kawasaki and Yamaguchi 2013; van den Berg et al. 2023; Zhang and Luck 2008). For studies that employed the latter two methods, rewards were assigned to all targets in a single trial, and participants were incentivized to enhance their overall WM performance, which required an increase in the total amount of WM resources to improve performance. Since we observed a significant reward effect in the reward expectation method, this suggests that the total amount of WM resources for completing the task was boosted; therefore, memory performance for each item in a trial can be indiscriminately enhanced and the overall performance can be improved. However, when reward binding was applied, since rewards were only associated with certain targets, memory resources may be prioritized to these stimuli over the others. In other words, not only is the total amount of WM resources increased but the flexible allocation of these resources among items based on the distribution of rewards also leads to enhanced performance for the rewarded target to a greater extent (i.e., more resources are directed to the rewarded target and fewer resources to the unrewarded ones). Because the effect size of the reward binding method is larger than that of the reward expectation method, we speculate that improving the flexibility of resource reallocation contributes to enhancing WM performance. Taken together, our findings suggest that the beneficial effect of reward can be attributed to both increasing the total amount of WM resources and improving the flexibility of resource reallocation, and the effects of these two aspects may be accumulative.

However, we observed no significant difference in the reward effects between the subliminal reward method and the reward expectation method, which partially contradicts the theoretical framework proposed by Bijleveld et al. (2012). According to this model, individuals initially process subliminal reward information in rudimentary brain structures, such as the striatum (Childress et al. 2008), and task performance can be directly boosted by increasing mental efforts without requiring conscious awareness. Conversely, if rewards can be consciously perceived, higher-level cognitive functions are engaged, which allows facilitated processing and enables the development of complex strategic behavioral responses that further enhance performance (Dehaene et al. 1998). Contrary to this framework, our findings suggest that, at least for WM, the conscious adoption of additional strategies may not yield practical benefits for WM performance. Since for these two reward methods, the improvements in WM performance required an increase in the total amount of WM resources, our results indicate that the WM resources available may be primarily determined by neurophysiological constraints (Bays 2015; Lisman and Idiart 1995; Wei et al. 2012), not by conscious strategy adoption. It is worth noting that results for the subliminal reward method may lack reliability, with only four studies included and a fail-safe N equal to 59. Therefore, these findings require further validation.

In addition, secondary subgroup analysis showed that compared to reward binding paradigms, the difference in reward effects between non-monetary and monetary rewards widens under the reward expectation paradigms (see Table 3; the difference in effect size between the non-monetary and monetary reward conditions was 0.40 in the reward binding paradigm and 1.46 in the reward expectation paradigm), and the divergence mainly lies in the monetary condition (d = 1.03 for reward binding and 0.52 for reward expectation). By cross-referencing the other moderators, we think that the type of WM possibly modulates the differential effects observed in the monetary condition between the reward binding and reward expectation methods. Under the monetary condition of reward expectation, 10 out of 22 studies examined verbal WM, which has smaller reward effects than visual WM; and under the monetary condition of reward binding, 16 out of 17 studies examined visual WM, which typically has greater reward effects. This may contribute to the difference in reward effects between the two reward paradigms. Note that the number of studies on non-monetary rewards is relatively small, and the two studies on non-monetary rewards under reward expectation conditions happen to have relatively large effect sizes (1.74 and 2.26); therefore, the current results might not be robust.

**The type of WM, paradigms of WM, and outcome measures.** Another interesting finding is that improvements in visual WM due to reward are greater than those in verbal WM. The fail-safe N reveals robustness in the observed results. There are two potential interpretations. Firstly, visual WM predominantly relies on cortical processing of visual stimuli, while verbal WM engages verbal information processing that is related to multiple brain regions, including the left occipito-temporal and bilateral intraparietal cortexes (Dehaene and Cohen 2007). Consequently, rewards may have a smaller effect on verbal WM tasks due to the possible involvement and cooperation of multiple brain regions, while their effect on visual WM is more pronounced (Adcock et al. 2006). Secondly, it is due to the influence of WM experimental paradigms. In cases where memory materials are verbal, there are a total of six studies using the n-back paradigm, which has the smallest effect size out of the WM paradigms; whereas for visual WM, there is only one study. Additionally, in cases where memory materials are visual, there are 14 studies utilizing the recall paradigm, which has the largest effect size out of the WM paradigms; whereas for verbal WM, there are only two studies. Hence, the reward effect in visual WM is greater than in verbal WM.

As the experimental paradigms may confound the effect of the type of WM, the type of outcome measure may also modulate the effect since the type of outcome measure is closely contingent on the experimental paradigm used in the study. For visual WM, about half of the studies (21/37) used an outcome measure of accuracy (see secondary subgroup analysis), which has the largest effect size among all three measures examined. Therefore, we suggest that the type of WM, the experimental paradigms, and the outcome measure are three moderators that should be jointly considered when assessing the effect of reward on WM.

Overall, the effects of reward on all outcome measures are significant, with the impact on accuracy being larger than for error and reaction time. On the one hand, the enhancement across all outcome measures suggests that reward may universally improve WM performance regardless of the outcome measure tested. Reward elevates both accuracy and processing speed (reflected by RT), with no apparent speed–accuracy trade-off. On the other hand, the effect on RT is the smallest among these outcome measures, indicating variability among these measures. Notably, in a third of the studies included, reward did not significantly reduce RT. We suspect that the effect of reward on RT may be influenced by the specific experimental instructions provided. If participants were instructed to be both fast and correct/precise, participants may try to be more accurate and respond faster at the same time. Conversely, if they are instructed to prioritize accuracy or precision to receive the reward, they may be more cautious and respond more slowly. In the current study, since most of the studies did not provide detailed descriptions of the instructions applied, it was not examined as a moderator and thus heterogeneity may result from this confound.

**Participant group.** Consistent with previous studies (Rhodes and Katz 2017; Juckel et al. 2006), we found that special groups such as elderly people and schizophrenia patients experience fewer benefits from rewards. This result indicates that the reward effect may vary across different populations. Individuals in these special groups may lack flexibility in WM, rendering them unable to optimize resource allocation or adjust mnemonic strategies according to different reward conditions. On a neural level, both older adults and individuals with schizophrenia may be affected by dopaminergic abnormalities, resulting in reduced reactivity to reward (Li and Rieckmann 2014; Barch 2005; Nieoullon and Coquerel 2003). However, the number of studies on special groups is limited. There are only six studies with special groups in our meta-analysis and different types of participants (i.e., the elderly and schizophrenia patients) were mixed in one subgroup, which increased within-group heterogeneity, meaning that the results may not be reliable (see more discussion in Section 6).

## 6. Limitations

There are several constraints of the current analyses. First, the number of available studies is limited. Due to our stringent inclusion and exclusion criteria, fewer studies were eligible. Future studies may consider less stringent criteria, and more diverse moderator variables can be evaluated. Second, in the moderator analyses, there are large error variances for some subgroups that contained only a few eligible studies with a small sample size. Due to potentially low statistical power, we did not perform secondary subgroup analyses for these subgroups. Finally, due to the limitation of the number of available studies, we have combined elderly individuals and schizophrenia patients into a collective category labeled “others” as a comparative subgroup against the “healthy adults” subgroup. This is for practical reasons, and inherent variations may be embedded within the “others” population, thereby rendering the results not universally representative of all subgroups. More attention should be paid to special groups in the future.

Moreover, the method of meta-analysis by pooling data from many studies inevitably leaves out some research questions related to detailed experimental procedures available only in individual studies, for example, how the reward effects may be influenced by the order of items appearing in a memory sequence, e.g., the primacy effect and recency effect. We speculate that rewards can enhance the primacy and recency effects as items at these temporal locations are more sensitive indicators for diagnosis (Melin et al. 2022). In addition, a neurological study has shown that activities in reward-sensitive brain regions, e.g., the fusiform gyrus and orbital-frontal cortex, were largest 4–8 s after receiving a reward cue (May et al. 2004). It is possible that performance for memory items presented during this period could be better improved by rewards. These research questions require further exploration.

It is worth noting that the WM capacity, as reflected in memory performance, may be considered a personal trait. This is because memory capacity can be influenced by genetic factors, exhibiting relatively stable individual differences (Ilkowska and Engle 2010). Conversely, memory capacity can also display state-dependent variations, since it may be influenced by situational factors such as mood, fatigue, and sleep deprivation (Engle and Kane 2004; Schmeichel 2007). Therefore, the reward effects reported in this study might be influenced by such factors.

Finally, although it was not applied in our meta-analysis, modern measurement theories, such as the Rasch psychometric model and entropy-based Construct specification equations (Melin et al. 2022), can provide a more accurate explanation of the relationship between task performance and memory ability to take task difficulty into account. The included studies had response accuracies ranging between 70–90%, indicating that the task difficulty was appropriately set and could effectively measure participants’ WM performance. However, for individual studies concerning special groups, e.g., the most capable or least capable individuals, the modern measurement theory can be a valuable approach for improving the accuracy and validity of measurements.

## 7. Conclusions

In conclusion, in this meta-analytic study, we systematically investigated the effect of reward on WM performance and the possible moderators that can affect its effect. The results showed that reward enhanced WM performance robustly even when exceptionally large effect sizes were deleted. The type of reward, reward methods, type of WM, paradigm of WM, outcome measures, and participant group moderated the effect of reward on WM performance. This result provides solid evidence that reward improves WM performance and reveals possible mechanisms of the processing underlying these improvements.

## Figures and Tables

**Table 1:** Characteristics of Individual Studies.

Study (Author/Year)	Sample Size	Type of Reward	Reward Methods	Type of WM	Paradigm of WM	Outcome Measures	Participant Group	Effect Size (d)
Allen and Ueno (2018) Experiment 1	20	Non-monetary	Reward binding	Visual	Recall	ACC	Healthy adults	1.12
Allen and Ueno (2018) Experiment 2	20	Non-monetary	Reward binding	Visual	Recall	ACC	Healthy adults	1.34
Allen and Ueno (2018) Experiment 3	20	Non-monetary	Reward binding	Visual	Recall	ACC	Healthy adults	2.85
Allen and Ueno (2018) Experiment 4	20	Non-monetary	Reward binding	Visual	Recall	ACC	Healthy adults	2.56
Beck et al. (2010)	31	Monetary	Reward expectation	Verbal	Recognition	RT	Healthy adults	3.89
Belayachi et al. (2015)	18	Monetary	Reward expectation	Verbal	N-back	ACC, RT	Healthy adults	0.18
Capa and Bouquet (2018)	60	Monetary	Reward expectation, Subliminal reward	Verbal	Others	ACC	Healthy adults	1.46
Cho et al. (2018)	65	Monetary	Reward binding	Visual	Delay estimation	Error	Healthy adults, Others	0.79
Cho et al. (2022)	33	Monetary	Reward binding	Visual	Delay estimation	Error	Healthy adults	0.68
Choi et al. (2015)	26	Monetary	Reward expectation	Visual	Recognition	ACC	Healthy adults	1.38
Cohen et al. (2019)	20	Monetary	Reward expectation	Verbal	Recognition	ACC	Healthy adults	0.99
Di Rosa et al. (2019)	21	Monetary	Reward expectation	Visual	Recognition	ACC, RT	Others	0.24
Gaillard et al. (2019)	23	Monetary	Reward expectation	Visual	Change detection	ACC, RT	Healthy adults	0.45
Gaillard et al. (2020)	32	Monetary	Reward expectation	Visual	Change detection	ACC, RT	Healthy adults	0.30
Grogan et al. (2022) Experiment 1	30	Monetary	Reward expectation	Visual	Delay estimation	ACC, RT	Healthy adults	0.86
Grogan et al. (2022) Experiment 4	30	Monetary	Reward binding	Visual	Delay estimation	ACC, RT	Healthy adults	0.98
Hager et al. (2015)	56	Monetary	Reward expectation	Verbal	N-back	ACC, RT	Healthy adults, Others	−0.01
Hammer et al. (2015)	34	Monetary	Reward expectation	Verbal	N-back	ACC	Healthy adults	0.84
Heitz et al. (2008)	30	Monetary	Reward expectation	Verbal	Others	ACC, RT	Healthy adults	0.52
Heuer and Schubö (2018) Experiment 1B	14	Monetary	Reward binding	Visual	Recognition	ACC, RT	Healthy adults	1.17
Heuer and Schubö (2018) Experiment 2	24	Monetary	Reward binding	Visual	Change detection	ACC	Healthy adults	1.15
Hinault et al. (2019)	19	Monetary	Reward binding	Visual	Others	ACC, RT	Healthy adults	1.88
Infanti et al. (2015)	30	Monetary	Reward binding	Visual	Recall	ACC	Healthy adults	2.85
Jiang (2013) Experiment 1	20	Non-monetary	Reward binding	Visual	Recall	ACC	Healthy adults	1.31
Jiang (2013) Experiment 2	20	Non-monetary	Reward binding	Visual	Recall	ACC	Healthy adults	1.13
Jimura et al. (2010)	31	Monetary	Reward expectation	Verbal	Recognition	RT	Healthy adults	1.58
Kawasaki and Yamaguchi (2013)	14	Monetary	Reward expectation	Visual	Change detection	ACC	Healthy adults	0.39
Klink et al. (2017) Experiment 1	10	Monetary	Reward binding	Visual	Delay estimation	Error	Healthy adults	3.23
Klink et al. (2017) Experiment 2	8	Monetary	Reward expectation	Visual	Delay estimation	Error	Healthy adults	−0.02
Lin and Fougnie (2022) Experiment 1	56	Monetary	Reward binding	Visual	Delay estimation	Error	Healthy adults	1.04
Lin and Fougnie (2022) Experiment 2	56	Monetary	Reward binding	Visual	Delay estimation	Error	Healthy adults	1.52
Magis-Weinberg et al. (2019)	50	Monetary	Reward expectation	Verbal	Recognition	ACC, RT	Healthy adults	0.50
Manga et al. (2020)	146	Monetary	Reward expectation	Visual	Delay estimation	Error, RT	Healthy adults, Others	0.02
Morey et al. (2011) Experiment 1	32	Monetary	Reward binding	Visual	Change detection	ACC	Healthy adults	0.82
Morey et al. (2011) Experiment 2	30	Monetary	Reward expectation	Visual	Change detection	ACC	Healthy adults	0.67
Morey et al. (2011) Experiment 3	24	Monetary	Reward binding	Visual	Change detection	ACC	Healthy adults	1.08
Sanada et al. (2013)	12	Monetary	Reward expectation	Visual	Change detection	ACC	Healthy adults	2.16
Sandry and Ricker (2020) Experiment 1	29	Non-monetary	Reward binding	Visual	Recognition	ACC, RT	Healthy adults	1.06
Sandry and Ricker (2020) Experiment 2	31	Non-monetary	Reward binding	Visual	Recognition	ACC, RT	Healthy adults	0.95
Sandry and Ricker (2020) Experiment 3	30	Non-monetary	Reward binding	Visual	Recognition	ACC, RT	Healthy adults	1.43
Savine et al. (2010) Experiment 2	30	Monetary	Reward expectation	Verbal	Recognition	ACC, RT	Healthy adults	0.24
Szatkowska et al. (2008)	16	Monetary	Reward expectation	Verbal	N-back	ACC, RT	Healthy adults	0.00
Taylor et al. (2004)	12	Monetary	Reward binding	Visual	Recognition	ACC, RT	Healthy adults	0.47
Thornton et al. (2007)	102	Monetary	Reward expectation	Visual	N-back	ACC	Healthy adults, Others	0.00
Thurm et al. (2018)	44	Monetary	Reward binding	Verbal	N-back	ACC, RT	Healthy adults, Others	0.97
Tian (2020) Experiment 1	24	Non-monetary	Reward binding	Visual	Recall	ACC	Healthy adults	2.12
Tian (2020) Experiment 2	24	Non-monetary	Reward binding	Visual	Recall	ACC	Healthy adults	2.35
Xu et al. (2022)	34	Monetary	Subliminal reward	Verbal	N-back	ACC, RT	Healthy adults	1.04
Zedelius et al. (2012) Experiment 1	41	Monetary	Subliminal reward	Verbal	Recall	ACC	Healthy adults	0.50
Zedelius et al. (2012) Experiment 2	33	Monetary	Subliminal reward	Verbal	Recall	ACC	Healthy adults	0.94
Zhang (2021) Experiment 1	26	Non-monetary	Reward expectation	Visual	Recall	ACC	Healthy adults	1.74
Zhang (2021) Experiment 2	30	Non-monetary	Reward expectation	Visual	Recall	ACC	Healthy adults	2.26
Zheng et al. (2022) Experiment 1	56	Monetary	Reward binding	Visual	Recall	ACC	Healthy adults	0.55
Zheng et al. (2022) Experiment 2	56	Monetary	Reward binding	Visual	Recall	ACC	Healthy adults	0.74
Zhou et al. (2021)	20	Monetary	Reward binding	Visual	Recall	ACC	Healthy adults	1.22

**Table 2:** Main results.

	No. of Studies	Sample Size	Cohen’s d	Standard Error	*Z*	*p*	95% CI	Heterogeneity			Fail-Safe N
								*Q*	*p*	*I* ^2^	
**Overall**	51	1711	0.93	0.08	11.71	<0.001	[0.77, 1.08]	314.88	<0.001	84.12	9362
**Moderator analysis**											
Type of reward											
Monetary	39	1361	0.78	0.08	9.61	<0.001	[0.62, 0.94]	210.23	<0.001	81.92	4440
Non-monetary	10	254	1.62	0.18	9.20	<0.001	[1.28, 1.97]	30.66	<0.001	70.65	713
Reward methods											
Reward binding	23	703	1.10	0.10	11.30	<0.001	[0.91, 1.29]	79.75	<0.001	72.41	2581
Reward expectation	28	1008	0.82	0.12	7.00	<0.001	[0.59, 1.05]	226.72	<0.001	88.09	2307
Subliminal reward	4	168	0.72	0.23	3.16	0.002	[0.27, 1.17]	18.15	0.006	83.47	59
Type of WM											
Visual	37	1150	1.05	0.10	11.15	<0.001	[0.87, 1.24]	209.82	<0.001	82.84	5712
Verbal	14	561	0.62	0.14	4.58	<0.001	[0.36, 0.89]	84.28	<0.001	84.58	433
Paradigm of WM											
N-back	7	300	0.43	0.19	2.25	0.025	[0.05, 0.80]	37.20	<0.001	83.87	46
Change detection	6	136	0.94	0.18	5.40	<0.001	[0.60, 1.29]	13.34	0.002	62.51	118
Recognition	13	349	0.79	0.13	5.97	<0.001	[0.53, 1.05]	56.10	<0.001	78.61	477
Recall	10	306	1.23	0.19	6.40	<0.001	[0.85, 1.60]	50.51	<0.001	82.18	508
Delay estimation	8	352	0.78	0.18	4.41	<0.001	[0.43, 1.13]	41.82	<0.001	83.26	240
Others	3	109	1.24	0.40	3.07	0.002	[0.45, 2.03]	15.82	<0.001	87.36	52
Outcome measures											
Error	8	423	0.75	0.16	4.60	<0.001	[0.43, 1.08]	43.06	<0.001	83.75	250
ACC	42	1257	0.95	0.09	10.88	<0.001	[0.78, 1.13]	241.85	<0.001	83.05	6265
RT	20	690	0.69	0.14	5.08	<0.001	[0.42, 0.96]	161.75	<0.001	88.25	806
Participant group											
Healthy adults	49	1452	0.95	0.08	11.53	<0.001	[0.79, 1.11]	290.38	<0.001	83.47	8503
Others	6	225	0.33	0.16	1.99	0.047	[0.01, 0.65]	30.26	<0.001	83.48	23

**Table 3:** Secondary subgroup analysis.

	No. of Studies	Sample Size	Cohen’s d	Standard Error	*Z*	*p*	95% CI	Heterogeneity			Fail-Safe N
								*Q*	*p*	*I* ^2^	
**Monetary reward**											
Outcome measures											
Error	8	418	0.86	0.20	4.25	<0.001	[0.46, 1.26]	54.02	<0.001	87.04	247
ACC	19	628	0.88	0.12	7.51	<0.001	[0.66, 1.12]	90.71	<0.001	80.16	1230
RT	15	541	0.48	0.14	3.54	<0.001	[0.21, 0.74]	95.86	<0.001	85.40	227
**Visual WM**											
Outcome measures											
Error	7	415	0.79	0.17	4.71	<0.001	[0.46, 1.12]	41.77	<0.001	85.64	252
ACC	21	524	1.24	0.12	9.96	<0.001	[1.00, 1.49]	81.13	<0.001	75.35	2006
RT	10	355	0.75	0.22	3.41	0.001	[0.32, 1.18]	97.27	<0.001	90.76	170
**Reward binding**											
Type of reward											
Monetary	15	469	1.03	0.08	13.40	<0.001	[0.88, 1.19]	22.85	0.063	38.73	1155
Non-monetary	8	198	1.53	0.19	7.88	<0.001	[1.15, 1.91]	22.94	0.002	69.48	400
**Reward expectation**											
Type of reward											
Monetary	20	724	0.52	0.10	5.08	<0.001	[0.32, 0.72]	91.78	<0.001	79.30	512
Non-monetary	2	56	1.98	0.26	7.71	<0.001	[1.48, 2.49]	1.43	0.232	29.98	--

## Data Availability

Data is available upon request.
